# Multiple Actin Isotypes in Plants: Diverse Genes for Diverse Roles?

**DOI:** 10.3389/fpls.2012.00226

**Published:** 2012-10-12

**Authors:** Kateřina Šlajcherová, Jindřiška Fišerová, Lukáš Fischer, Kateřina Schwarzerová

**Affiliations:** ^1^Faculty of Science, Department of Experimental Plant Biology, Charles University in PraguePrague, Czech Republic

**Keywords:** isotype, isovariant, actin, actin binding protein, expression, regulation

## Abstract

Plant actins are encoded by a gene family. Despite the crucial significance of the actin cytoskeleton for plant structure and function, the importance of individual actin isotypes and their specific roles in various plant tissues or even single cells is rather poorly understood. This review summarizes our current knowledge about the plant actin gene family including its evolution, gene and protein structure, and the expression profiles and regulation. Based on this background information, we review mutant and complementation analyses in *Arabidopsis* to draw an emerging picture of overlapping and specific roles of plant actin isotypes. Finally, we examine hypotheses explaining the mechanisms of isotype-specific functions.

## Introduction

Actin is an essential protein that is expressed throughout the plant body in many distinct isotypes (the term *isotype* is commonly used for closely related proteins, and *isoform* or *isovariant* are often used as synonyms to *isotype*). The structure, regulation of expression and expression patterns of actin genes, and the roles of the proteins have been extensively researched, yet fundamental questions remain. For example, what are the factors that account for the occurrence of different actin isotypes and what are their functions? Although the issue of diverse roles of actin isotypes has been partly addressed at the tissue level, information at the cellular level is scarce. To better understand the existence and role of multiple actin isotypes in plants, this review first summarizes what is known about the structure of actin genes, evolution of the gene family, and expression regulation. Further, we examine the current evidence for diverse and overlapping roles of actin isotypes and the molecular mechanisms determining the diverse roles.

## Multiple Actin Genes and Their Structure

The numbers of actin genes in various plant species examined so far indicate that this gene family is quite variable. The total number of actin family members present in public databases (National Center for Biotechnology Information, NCBI^1^, Plant Genome Database^2^, and Phytozome^3^; Table 1) partially reflects the complexity of the respective organism. Red algae usually contain two actin isotypes, a conserved one and a variable one (Le Gall et al., 2005; Wu et al., 2009) and two actin isotypes are found in the green algae *Chlamydomonas* and *Volvox*. More complex plants, including many mono- and dicots as well as the gymnosperm *Pinus taeda* (Schwarzerova et al., 2010) and the moss *Physcomitrella patens* usually contain 7–11 actin isotypes (Meagher et al., 1999a; Zhang et al., 2010). Surprisingly, only three actin isotypes have been found in *Selaginella moellendorffii* and four in *Medicago truncatula*, although the number of identified isotypes may not be final. A surprisingly large family of actin isotypes has been identified in maize (21 members), although some of them may represent pseudogenes. Similar situation may occur in other species as well, so the actual number of actin isotypes can differ from the values identified so far (Table 1). For example, exceptionally high number of actin genes was identified in *Petunia*, where more than 100 members of actin gene family arose probably through local duplication of actin gene subfamilies (Baird and Meagher, 1987). Nevertheless, the exact number of active actin genes and pseudogenes in petunia is not known.

**Table 1:** **Total number of actin genes in organisms representing major plant groups**.

	Organism	Number of actin genes
Algae	*Volvox carteri* and ***Chlamydomonas reinhardtii***	2
	*Porphyra purpurea* and *Palmaria* *palmata*	2–3
Moses	***Physcomitrella patens***	10
Lycopsida	***Selaginella moellendorffii***	2–3
Gymnosperms	*Pinus taeda*	10
Monocots	***Sorghum bicolor***	10
	***Zea mays***	21
	***Oryza sativa***	8–10
Dicots	*Vitis vinifera*	6
	***Populus trichocarpa***	8–9
	*Manihot aesculenta*	11
	***Arabidopsis thaliana***	8 (10)
	***Medicago truncatula***	4

*The numbers were estimated using public databases (NCBI, Plant Genome Database, and Phytozome). Organisms with a known genome sequence are highlighted in bold. For *Arabidopsis*, the total number of actin genes including probable pseudogenes is shown in parentheses*.

In contrast to plants, most metazoans generally contain fewer than 10 functional actin genes (Bhattacharya et al., 2000). For example, there are six actin genes encoding six distinct actin isotypes in birds and mammals (Perrin and Ervasti, 2010). Therefore, the plant actin gene family seems to be rather more diversified and complex compared with other multicellular organisms. Since the number of plant actin genes increases along with increase in tissue complexity, one might hypothesize that more complex plant organisms with complex tissues need more diversified actins. However, other factors are also likely to contribute to the evolution of the complex actin gene family in plants. Bhattacharya et al. (2000) hypothesized that rather than reflecting the development of complex tissues, the diversification of the actin gene family in angiosperms may have originated in combination of relaxed constraints on actin evolution, frequent gene duplication events through polyploidization or hybrid speciation, and other factors (Bhattacharya et al., 2000).

**Figure 1:**
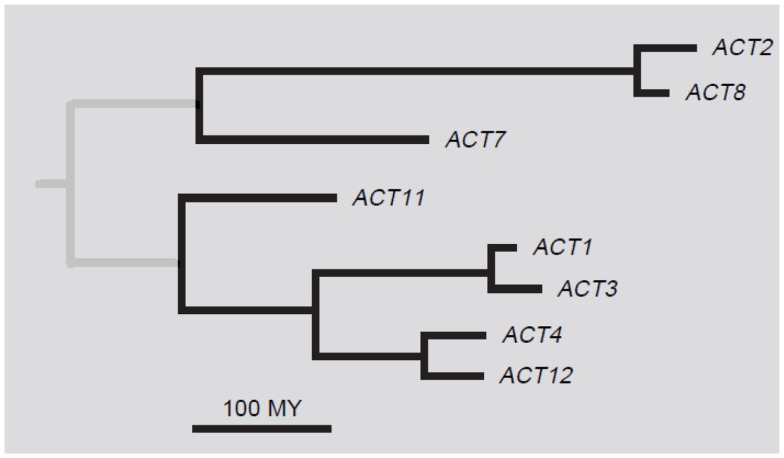
**A phylogenetic tree of *Arabidopsis thaliana* actins**. Adapted with permission from Meagher et al. (1999b).

The most complete information about a plant actin gene family is available for *Arabidopsis thaliana*. Here, the family contains 10 actin genes (*ACT*), eight of which encode functional proteins and two likely pseudogenes (*ACT5* and *ACT9*). According to protein sequences, the actin family is divided into two major classes: vegetative and reproductive. The eight protein isotypes can be further divided into five subclasses: two single-member subclasses ACT7 and ACT11, and three pairs of closely related proteins, namely, ACT2/8 in the vegetative class and ACT1/3 and ACT4/12 in the reproductive class (Figure 1). The two major classes and the three pairs of the closely related paralogs show unique expression patterns congruent with the phylogenetic tree (McDowell et al., 1996b). *ACT5* and *ACT9* are considered as pseudogenes, because they diverged from other actins considerably and no expression of these genes was detected in experiments of McDowell et al. (1996b). However, the chip expression analysis by Genevestigator^4^ showed that both *ACT5* and *ACT9* are expressed, especially in endosperm. Possible involvement of these two genes in endosperm development needs further analysis.

**Figure 2:**
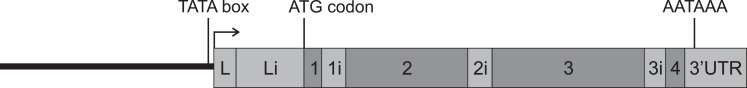
**Schematic structure of a general plant actin gene**. Translated regions are shown in dark gray and transcribed regions in light gray; a thick black line indicates regulatory region. L and Li are leader exon and leader intron, respectively. Numbers 1–4 are exons and numbers 1i–3i are introns. Transcription start and translation start are indicated by an arrow and ATG, respectively, and AATAAA is a polyadenylation element.

Studies of actin genes in plants, mainly *Arabidopsis*, revealed that the gene structure is quite conserved. Generally, the coding sequence consists of four exons and three introns, preceded by one non-coding leader exon and an intron located in the 5′ UTR (Figure 2; McDowell et al., 1996b). This scheme is largely universal, although there are few exceptions. In *Arabidopsis* *ACT2*, the intron 2 is missing (McDowell et al., 1996b). Some actin genes, such as *ACT2* and *ACT8*, lack the consensus polyadenylation element AATAAA and polyadenylation is instead controlled by other sequences (An et al., 1996b). Other polyadenylation sequences often occur, such as the five polyadenylation sequences in *ACT11* (Huang et al., 1997), four in *ACT1* and three in *ACT3* (An et al., 1996a). It has been proposed that these alternative sequences may represent a safety mechanism to avoid mRNA degradation through RNA interference, which can be induced by un-polyadenylated transcripts under strong expression (Luo and Chen, 2007). Multiple transcription initiation sites also exist, such as the three sites in *ACT1* (An et al., 1996a) and two in *ACT7* (McDowell et al., 1996b). Other kinds of structural variability have been identified in plant species other than *Arabidopsis*. In *Gossypium hirsutum* gene *GhACT8*, glutamine Gln151 (codon CAG) is inserted 4 bp upstream from the exon-intron 2 junction so that the intron 2 is located at amino acid residue 153 instead of the usual residue 152, which is common in other cotton actin genes (Li et al., 2005). In the hemiparasitic plant *Striga asiatica*, actin gene *SAA2* lacks intron 2, which is the same intron that is missing in *Arabidopsis ACT2*. Also, *S. asiatica* actin gene *SAA3* has a unique sequence within intron 2, indicating that this gene lost its function and turned into a pseudogene (Florea and Timko, 1997). Taken together, it is likely that this variability is mostly caused by a need for differential regulation of expression, which could be achieved for instance by distinct post-transcriptional processing.

## Actin Gene and Protein Sequences

While the exon/intron structure of actin genes is well conserved, the nucleotide sequences of individual isotypes are somewhat more distinct from each other. Even though the isotypes in the paralog pair ACT1/3 are identical and those in the closely related paralog pairs ACT 2/8 and ACT4/12 differ in only one amino acid substitution, the nucleotide-coding sequences within these paralog pairs differ by 9–12%. The difference at the nucleotide level within the family reaches 22%, only about twice as much as the difference between the closest paralogs. Overall, at the amino acid level the eight functional actin isotypes in *Arabidopsis* differ by 0.3–8.8%, with the most distant isotypes ACT2 and ACT4 differing in 33 amino acids.

Given the high number of silent nucleotide substitutions even in the almost identical proteins in the three closely related paralog pairs (McDowell et al., 1996b), the actin genes must have had a long evolutional history. Yet, their protein sequences are markedly conserved. The multitude of changes at the nucleotide level in contrast to just a few changes at the amino acid level suggests that the selection pressure on the structure of actin protein isotypes must have been extraordinarily strong. Even a single amino acid substitution has a potential to modulate the protein function. Nevertheless, the *Arabidopsis* actins have substitutions of a charged residue or proline at 11 positions that are distributed over the surface of the protein molecule, with nine of the substitutions potentially having significant effect on the biochemical character of the protein (McDowell et al., 1996b). This variability can be expected to play a role in actin–actin or actin–actin binding protein (ABP) interactions.

Interestingly, the differences among individual actin isotypes in plants are greater than those in animals. There are almost as many substitutions in charged amino acid residues between any two subclasses in *Arabidopsis* as in the entire actin family in humans (Huang et al., 1997). Vertebrate cytoplasmic and muscle actins differ in about 5% of the amino acid sequence; these are represented by only a few changes in charged residues, showing much less variability than plant actins (An et al., 1996b). Also, vertebrate cytoplasmic and muscle actins differ in their isoelectric points by only 0.3 pH units, whereas *Arabidopsis* vegetative and reproductive actin isotypes differ by 0.7 pH units (Meagher et al., 1999a). Moreover, the amino acid substitutions in plant actins are more frequently located on the surface of the protein molecule compared with those in the animal actins (Kandasamy et al., 2007).

## Evolution of the Actin Gene Family

Green algae contain only one or two actin genes, whereas the number of actin genes increases in land plants (Bhattacharya et al., 2000, Table 1). Molecular studies have revealed high complexity of the angiosperm actin gene family, which is broadly divided into a vegetative and a reproductive clade. The phylogenetic analysis suggested that vegetative actins represent a more primitive group of actins that share common ancestry with fern actins. It has been suggested that the reproductive group of actins has probably evolved from the vegetative actins, perhaps reflecting the need of new functions in newly evolved structures (An et al., 1999).

The moment of separation of individual actin isotypes can be estimated according to the character and number of changes in the sequences of the actin genes. A phylogenetic analysis of *Arabidopsis* actins suggested that vegetative and reproductive actin gene groups diverged about 350–400 million years (MY) ago (Gilliland et al., 1998). This corresponds to the time when plants left the water environment and embarked on land and developed reproductive structures. ACT11 diverged from the rest of the reproductive isotypes about 220 MY ago when the primitive and advanced gymnosperms diverged (Kandasamy et al., 1999). The other subclasses diverged about 200 MY ago (An et al., 1996a; Gilliland et al., 1998; Kandasamy et al., 2009). This time coincides with the evolution of flowering plants that arose about 130–217 MY ago (Specht and Bartlett, 2009; Smith et al., 2010). These data suggest that new actin isotypes did not develop randomly, but that their origin reflected the need for new proteins and functions. The coincidence of the divergence of the actin gene family with the divergence of plant species may be similarly followed in *Populus* (Zhang et al., 2010).

## Regulatory Sequences and Regulation of Expression

The expression of actin isotypes is tightly regulated. In *Arabidopsis*, regulation at the transcriptional as well as post-transcriptional level was described. Transcriptional regulation regions are located mainly upstream of the TATA box, while post-transcriptional regulation regions lie in the leader exon/intron in the 5′ UTR. Individual *Arabidopsis* actin isotypes differ in their regulatory elements and the elements themselves differ in their roles in different parts of the plant. Vitale et al. (2003) mutated various parts of a conserved domain located immediately before the TATA box in *ACT1* and found that different parts of the domain influenced the expression of *ACT1/3* to various extent in different parts of the plant. *ACT7* contains various hormone-responsive conserved elements located upstream of the TATA box, including auxin-, abscisic acid-, gibberellin-responsive elements, and others. These elements enable this actin to react to hormonal stimuli, which is a unique ability among actins (McDowell et al., 1996a). Similar elements are expected to be present in the *ACT7* homolog in *Malva pusilla* because this isotype is induced by an interaction of the plant with pathogenic fungi (Jin et al., 1999).

The leader exon/intron in the 5′ UTR region appears to play a crucial role in the regulation of actin expression. In the 5′ UTR of the *ACT2/8* pair there are 15 conserved elements of a minimal length of 6 bp, which are also shared by a related actin isotype in *P. patens* (An and Meagher, 2010). This high degree of conservation suggests a common and important role of the region. Although disruption of any of these elements did not result in dramatic changes in the expression of a reporter beta-glucuronidase (GUS) fused to the modified 5′ UTR, deletion of the entire leader intron led to heavily impaired expression (An and Meagher, 2010). The function of the 5′ UTR as a regulatory element was also described for *ACT7* (Gilliland et al., 2003) and *ACT1* (Vitale et al., 2003), suggesting that the leader intron plays a crucial role in actin expression and that this regulation may be common to all actin isotypes. Multiple questions emerge, not least of which is whether the 5′ UTR regions are interchangeable or specific? Could the 5′ UTR region determine the specific expression pattern or the function of some of the actin isotypes? Experiments of Vitale et al. (2003) revealed that replacing the reproductive *ACT1* leader intron with the vegetative *ACT2* leader intron (*ACT1* and *ACT2* are evolutionarily distant actins with antagonistic expression) led to the preservation of the *ACT1* expression, but only in vegetative tissues and not in mature pollen (see Table 2). Thus, it is indeed possible that the leader intron could be an important factor in determining the expression pattern of actin isotypes (Vitale et al., 2003). Although this hypothesis still lacks experimental support, it is a signpost in complex matters that we are only beginning to understand.

**Table 2:** **Expression patterns of actin isotypes and their possible function**.

	Expression pattern	Possible roles	Reference
ACT1	R tips, LatR Pr, SA, VT, young L, Tr, St MP: young buds, floral organs Pr, not mature organs. Strong in P, PT	P germination, PT growth, transport of vesicles and generative nuclei to tip in PT + vesicle fusion + VT differentiation and secondary wall deposition. Positioning and formation of cell division planes in meristems, preprophase band formation	An et al. (1996a)
ACT3	Same as ACT1, only more in L, less in P	Same as ACT1	An et al. (1996a)
ACT4	Young VT, only weak in older VT	Same as ACT1, except for cell division functions	Huang et al. (1996a)
	MP: weak in anthers and microspores but increased during P development. Extremely high levels in trinucleate P and PT.	
ACT12	VT same as ACT4 + pericycle during latR Pr development (ring-like expression) + R cap	Same as ACT1 + graviperception + vesicle trafficking and fusion in R cap + the cell division functions not in meristems, but in LatR initiation	Huang et al. (1996a)
ACT11	Same as ACT4 + in young L everywhere + SA + etiolated hypocotyl everywhere MP: flower primordia and young buds, then Pi only. Extremely high in trinucleate P, PT. Strong, ubiquitous in O	Same as ACT1 + secretion on Sti, helping PT to grow through the Sty + O development, asymmetric partitioning + rapid cell elongation	Huang et al. (1997)
ACT2	Whole plant, especially SA. Not in hypocotyl MP: sepals and Pi. Before anthesis decreases in ovary, increases in Sti, Sty. Connective tissues between pollen sacs, but not in anther or P	Universal processes (cytoplasmic streaming, cytoarchitecture around nucleus, preprophase band)	An et al. (1996b)
ACT8	Same as ACT2, only weaker and in subset of tissues. Different distribution in R apex, not in Pi	Same as ACT2	An et al. (1996b)
ACT7	Aleurone layer of seed, whole seedling. Emerging R, L and their Pri, in older L only Tr, St, VT. All patterns changeable under phytohormone treatment	Links phytohormones with developmental processes	McDowell et al. (1996a) and Kandasamy et al. (2001)
	MP: all expanding developing tissues Callus, hormone-treated tissues	Callus formation	

*The expression patterns were obtained from 7- to 10-day-old seedlings, 3-week-old juvenile plants, and 6-week-old mature, flowering plants using actin-GUS fusions; data from total RNA analysis and RT-PCR generally confirmed the results. Reproductive isotypes are shown on a white background and vegetative isotypes on a gray background; thick lines indicate subsets of isotypes subclasses. *ACT5* and *ACT9* were not included as likely pseudogenes. MP, mature plant; R, root; LatR, lateral root; Pr, primordia; SA, shoot apex; VT, vascular tissue; L, leaf; Tr, trichomes; St, stomata; P, pollen; PT, pollen tubes; Pi, pistil; O, ovule; Sti, stigma; Sty, style*.

Translational regulation of actins has been far less explored. Evidence of specific translation regulation in pollen and some other tissues was presented by An et al. (1996b). In their experiments, *ACT2* mRNA was present both in pollen and in vegetative tissues. However, the ACT2-GUS activity was detected in a high amount in nearly all vegetative tissues, but little or not at all in pollen. Similar data were shown for ACT8. Although *ACT8-GUS* transcript was relatively weaker but again present in a subset of tissues including pollen, the GUS activity itself was not found in pollen. These results suggested that the expression regulation in pollen is different from other tissues and may include translational level of regulation (An et al., 1996b).

An intricate set of regulatory elements enables finely regulated and complex expression patterns of actin isotypes. Highly controlled regulation is essential for unique expression patterns and for expected mutual collaboration between the isotypes. Moreover, some regulatory elements are conserved, although many of them are markedly divergent. The variety of regulatory elements indicates that the expression of actin isotypes is under the control of diverse mechanisms. It is a challenge for future research to uncover the regulatory elements and their precise roles in the regulation of actin expression.

## Expression Patterns of Actin Isotypes

As mentioned above, individual plant actins show different expression patterns, congruent with their evolutionary relationships. Generally, the *Arabidopsis* reproductive actins (ACT1/3, ACT4/12, and ACT11) tend to be expressed in reproductive tissues, whereas the vegetative actins (ACT2/8 and ACT7) are expressed in vegetative ones. Specifically, the ACT1/3 pair is expressed in developing floral meristem, the emerging carpel and ovules, and in mature pollen (An et al., 1996a; Meagher et al., 1999b). ACT4/12 are expressed in young vascular tissues, tapetum, and developing and mature pollen, whereas ACT12 expression was detected also in the pericycle during lateral root initiation and early development (Huang et al., 1996a). ACT11 is the most distinct reproductive actin, with strong expression in rapidly elongating tissues, floral organ primordia, mature pollen, ovule, embryo, and endosperm (Huang et al., 1997). ACT2 was expressed in all vegetative tissues and no expression was detected for example in gynoecia or pollen sacs, whereas ACT8 expression was weaker and detectable only in a subset of tissues expressing ACT2 (An et al., 1996b). ACT7 was expressed in all young vegetative tissues, hypocotyls, and seed coat (McDowell et al., 1996a). Nevertheless, the expression patterns overlap to a certain extent (Meagher et al., 1999b). The relevant data on the expression of *Arabidopsis* actin isotypes are summarized in Table 2.

Developmental changes in the dynamics of actin isotypes certainly play an important role in the complex process of embryogenesis. We found that *Picea abies* actin isotype *Pa1* was expressed in the whole somatic embryo, while isotypes *Pa2-4* were expressed predominantly in the suspensor and their expression decreased as the suspensor degenerated later in development (Schwarzerova et al., 2010). These results are consistent with the hypothesis that multiple actin isotypes are expressed simultaneously in plant cells, but also support the notion that the isotype composition is specifically tuned during various stages of plant development. Most information about isotype composition and dynamics has been obtained using the angiosperm model *A. thaliana*, although no detailed data are available on differential expression of actin isotypes during the embryogenesis. Therefore, results from experiments in various plants are difficult to compare and more plant models are urgently needed to better understand the diversity, regulation, and the role of actin isotypes in plants.

## Cellular Localization Pattern of Actin Isotypes

In animal cells, the localization of muscle and cytoplasmic actin isotypes in muscle and non-muscle cells was described in several studies, even though with somewhat conflicting results (Perrin and Ervasti, 2010). It seems that a high structural similarity among actin proteins is a significant barrier for localization studies attempting to distinguish individual isotypes immunochemically. Similarly in plants, only scarce information is available on the distribution and cooperation of various actin isotypes in cytoplasmic actin filaments. Abundant descriptions exist about the various characteristics of the actin network for specific plant cell types including pollen tubes, root hairs, and leaf epidermal cells (reviewed in Staiger et al., 2000). Nevertheless, the role of individual actin isotypes within defined sets of filaments or within specific cellular compartment remains largely elusive. In this respect, a study by Kandasamy et al. (2010) showed differential localization of ACT7 and ACT2/8 within the nucleus, with ACT7 being more concentrated in nuclear speckles and nucleolus than ACT2/8. However, this study is rather solitary and more data are needed to uncover the subcellular characteristics and behavior of specific actin isotypes.

## Diverse Roles of Actin Isotypes

In the preceding sections we attempted to summarize existing knowledge about plant actin genes and proteins. The differences among plant actin isotypes at the protein level are higher and amino acid substitutions are more frequent than those in animal actins, suggesting that individual actin isotypes may have specific roles in plant tissues. The expression analysis likewise suggested the existence of vegetative and reproductive actins. However, is there direct experimental evidence that plant actin isotypes have specific, non-overlapping functions? What is the molecular mechanism of diverse actin functions in the cell? And importantly, is there biochemical evidence for diverse roles of actin isotypes?

Studies in animals suggest that actin isotypes fulfill specific roles in these organisms, with some overlapping functions. Substitution of a muscle actin isotype with cytoplasmic actin in *Drosophila* results in a flightless phenotype because the cytoplasmic actin is not able to substitute for muscle actin (Brault et al., 1999). In mammals, four actin genes encode four distinct skeletal actin isotypes (α-skeletal, α-cardiac, α-smooth, and γ-smooth actin), whereas two other genes encode two cytoplasmic actin isotypes (β- and γ-cytoplasmic actin). Mammalian cytoplasmic and muscle actin isotypes perform rather specialized functions. Nevertheless, at least two skeletal actin isotypes (α-skeletal and α-cardiac actin) overlap in their expression and function in knock-out mice, thus indicating both overlapping and distinct functions (Perrin and Ervasti, 2010).

In plants, the specific roles of individual actin isotypes were mostly studied by mutation analyses. In shoots, the vegetative actin isotype ACT2 comprises approximately 50% of the total actin protein and closely related ACT8 is much more weakly expressed, comprising 10–15% of the total actin protein. ACT7, a more divergent isotype, represents 40–45% of the total actins (Kandasamy et al., 2009). The ACT2 and ACT8 apparently play roles in root hair development in *Arabidopsis*. Aberrant root hairs were exhibited in several mutants, namely, missense *act2* mutants (Ringli et al., 2002), *act2-1* T-DNA insertional line null for ACT2, and *act8-2* T-DNA insertional mutant (Kandasamy et al., 2009). Since the total actin protein level in the mutants was often reduced, the phenotype could be partially related to decreased levels of actin in tissues. The aberrant root hair phenotype could be alleviated if ACT8 was overexpressed in *act2-1* mutants or ACT2 was overexpressed in *act8-2* mutants, suggesting the two isotypes are indeed mutually interchangeable in their roles in root hair development (Kandasamy et al., 2009).

Gilliland et al. (2002) reported that overexpression of *ACT7* using native regulatory sequences in transgenic plants, or ectopic expression of the *ACT1* coding region under the regulatory regions of *ACT2*, efficiently complemented *act2-1* loss-of-function mutant root hair phenotype. Kandasamy et al. (2009), however, reported only a partial suppression of the root hair phenotype in these plants and concluded that the ACT7 isotype is functionally distinct from ACT2/8 (Kandasamy et al., 2009). Indeed, mutants lacking ACT7 seem to have a distinct phenotype when compared with mutants lacking ACT2 or ACT8. In Gilliland et al. (2003) experiments, a T-DNA insertional mutant *act7-4* had 20-fold reduced levels of ACT7 protein and the total actin protein was reduced to 60–80% of the wild-type levels. In contrast to the *act2-1* loss-of-function mutant, which exhibited root hair phenotypes, *act7-4* mutants showed specific defects in germination, root growth control, and polarity control (Gilliland et al., 2003). However, the overexpression of ACT2 or ACT8 in transgenic plants fully suppressed the *act7-4* phenotype (Kandasamy et al., 2009). A specific function of ACT7, distinct from ACT2 and ACT8, was demonstrated in complementation experiments using double mutants. Generally, double mutants exhibited more severe defects when compared with single mutants, perhaps due to decreased total actin levels in tissues. Whereas a severe dwarfed phenotype in *act2-1 act7-4* mutant plants could be fully rescued by overexpressing ACT8, only a partial rescue of *act2-1 act8-2* phenotype was achieved in plants overexpressing ACT7 despite the fact that the total actin protein level in the rescued plants was elevated twofold compared with wild-type plants (Kandasamy et al., 2009). This again demonstrated partially overlapping functions of plant vegetative actins, and revealed that the ACT7 isotype has specific and distinct function.

Other studies showing functional non-equivalence of plant actin isotypes are also available. Ectopic expression of reproductive actin ACT1 in vegetative tissues of transgenic plants caused severe phenotypes including dwarfism and morphological abnormalities in most organs (Kandasamy et al., 2002). At the cellular level, extensive actin polymerization, bundling, and reorganization was detected in cells of vegetative tissues expressing the reproductive ACT1. By contrast, similar overexpression of a vegetative ATC2 had no effect (Kandasamy et al., 2002), suggesting that the reproductive actin ACT1 is functionally non-equivalent with ACT2, consistent with its specific role in reproductive tissues.

As mentioned above, phylogenetic analysis showed that plant vegetative actins represent a more primitive group of actins, whereas reproductive actins are more diverged. An exciting study of Kandasamy et al. (2012) suggests that the function of vegetative actins is indeed related to ancient actin functions which are also performed by the actin cytoskeleton in lower eukaryotes. The authors showed that protist actins from *Monosiga brevicollis*, *Acanthamoeba castellanii*, and *Coleochaete scutata*, which are close to the common ancestor of plant and animal actins, completely suppressed root phenotypes in *Arabidopsis* *act8-2 act7-4* double mutants. Interestingly, human cytoplasmic actin, but not human α-skeletal muscle actin, partially suppressed the phenotype as well (Kandasamy et al., 2012). These data indicate that during their independent evolution, plant vegetative and human cytoplasmic actins inherited from the protist primitive actins basic essential roles that are needed for their functioning in the cytoplasm. Since phylogenetic analyses showed that plant vegetative and human cytoplasmic actins are grouped into distinct ancient classes, Kandasamy et al. (2012) hypothesize that non-progressive sequence evolution occurred where ancient protein structures and functions, but not protein sequences, have been conserved, thus accounting for functional inter-changeability of protist and human actins in plants. Further research is needed to elucidate the basis of functional non-redundancy and overlaps of plant actin isotypes. It would be of particular interest to examine the functional inter-changeability of plant vegetative actins in protists and in animal models.

## Mechanism of Isotype-Specific Function

What is the molecular basis of the functional specificity between actin isotypes? As mentioned above, actins are highly conserved and differences between isotypes are very small. Nevertheless, actin–actin or actin–ABP interactions may still be affected by these differences, resulting in different phenotypes. Similarly to the family of actin isotypes, plant profilins likewise constitute a family where the isotypes are divided into vegetative and reproductive classes (Huang et al., 1996b). Therefore, a diverged clade of reproductive actins may be incapable of cooperating with a vegetative clade of ABPs. Indeed, an ectopic expression of reproductive actin ACT1 in vegetative tissues resulted in an altered phenotype, whereas co-expression of reproductive ABPs together with a reproductive actin in vegetative tissue restored a wild-type phenotype (Kandasamy et al., 2007).

A dominant-negative mutation in the *ACT2* gene was isolated in which a missense mutation resulted in an amino acid substitution at the surface of the protein molecule (Nishimura et al., 2003). The mutant plants showed a severe phenotype including defects in root development, whereas the phenotype of a loss-of-function *act2* mutant did not show most of the root developmental defects (Gilliland et al., 2002; Nishimura et al., 2003). Although the structural change in the protein molecule is small, the actin–actin or actin–ABP interactions may be affected and cause a mutant phenotype. Thus, even subtle differences in the sequence of the isotypes may affect the protein function and interactions.

Unfortunately, only limited information is available about the biochemical properties of individual actin isotypes. Recently, Bergeron et al. (2010) purified recombinant animal β- and γ-cytoplasmic actins and characterized their biochemical properties. Calcium-bound actins readily co-polymerized when mixed together. However, Ca-bound γ-cytoplasmic actin showed reduced dynamics in microfilament nucleation and elongation. Similar studies describing the biochemical properties of plant actins are missing.

## Conclusion

Available data suggest that plant actin isotypes have both overlapping and distinct functions. Complementation studies suggest a modified view of actins’ evolution. As suggested by Kandasamy et al. (2012), in spite of sequence divergence, protist actins and ancient groups of plant and animal actins may have retained the basic properties needed for their functioning in the cell. Divergent groups of actins, such as the plant reproductive actins or animal muscle actins, developed independently to fulfill specialized functions in the new structures that arose during evolution of the various groups of organisms, thus losing some of the basic properties.

Besides the generally high functional inter-changeability, some functional specialization can be detected even within the group of plant vegetative actins. Indeed, the relative proportions of actin isotypes with different biochemical properties may determine the characteristics of actin polymers. Theoretically it may be advantageous for a cell to express multiple isotypes with overlapping expression patterns, capable of mutual interactions, and cooperation with specific isotypes of ABPs. Using the analogy of steel as an alloy composed of multiple metals, an actin filament can be considered as an alloy composed of multiple actin isotypes (Perrin and Ervasti, 2010). Varying mixture of actins may tune the properties of the actin polymer, making it more readily adaptable to various specific functions (Meagher et al., 1999a). A more thorough characterization of biochemical properties of individual actin isotypes is needed to test the above hypotheses.

## Conflict of Interest Statement

The authors declare that the research was conducted in the absence of any commercial or financial relationships that could be construed as a potential conflict of interest.

## Footnotes

^1^www.ncbi.nlm.nih.gov

^2^www.plantgdb.org

^3^www.phytozome.net

^4^www.genevestigator.com
